# *QuickStats:* Age-Adjusted Rates of Drug Overdose Deaths Involving Heroin,* by Race/Ethnicity† — National Vital Statistics System, United States, 1999–2017

**DOI:** 10.15585/mmwr.mm6837a5

**Published:** 2019-09-20

**Figure:**
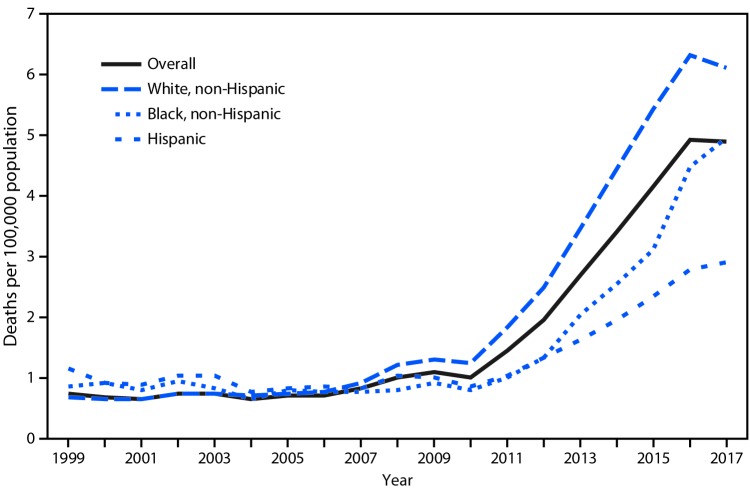
From 1999 to 2005, the overall age-adjusted rate of drug overdose deaths involving heroin in the United States remained stable at approximately 0.7 deaths per 100,000 population. The rate increased slightly from 0.7 in 2005 to 1.0 in 2010 and further increased to a high of 4.9 in 2016 and 2017. From 2010 to 2017, rates generally increased for each of the racial/ethnic groups shown, with the highest rates observed for non-Hispanic whites. In 2017, the rates were 6.1 for non-Hispanic whites, 4.9 for non-Hispanic blacks, and 2.9 for Hispanics.

**Source:** National Center for Health Statistics, National Vital Statistics System mortality data. https://www.cdc.gov/nchs/deaths.htm.

For more information on this topic, CDC recommends the following link: https://www.cdc.gov/drugoverdose/prevention/index.html.

